# Monitoring the Cerebral Oximetry Index Along With In-line Cardiopulmonary Bypass Parameters in a High-Risk Patient Undergoing Cardiac Surgery: A Case Report

**DOI:** 10.7759/cureus.40426

**Published:** 2023-06-14

**Authors:** Anna Gkiouliava, Despoina G Sarridou, Helena Argiriadou

**Affiliations:** 1 Department of Anaesthesiology, AHEPA University Hospital, Aristotle University of Thessaloniki, Thessaloniki, GRC

**Keywords:** complications, autoregulation, cerebral oximetry, cardiopulmonary bypass circuit, adult cardiac surgery

## Abstract

The quest to minimize the morbidity and mortality of patients undergoing cardiac surgery is ongoing. Impaired cerebral autoregulation and tissue malperfusion are linked with neurological complications. The cerebral oximetry index (COx) has been introduced as an index of cerebral autoregulation, while in-line monitoring enables the detection and prevention of metabolic disturbances during cardiopulmonary bypass (CPB).

This report presents the case of a 58-year-old female patient scheduled for aortic valve replacement under minimally invasive extracorporeal circulation (MiECC). Her medical history consisted of epilepsy, multiple ischemic strokes, heavy smoking, and brachiocephalic artery stenosis. We sought to investigate the limits of autoregulation and the role of metabolic indices of perfusion on COx. Mean arterial blood pressure (ABP), cerebral oximetry (rSO_2_), and in-line perfusion data during CPB were recorded at 10s intervals. The lower limit of autoregulation was 44mmHg on both sides and the upper limit was 98mmHg on the right and 107mmHg on the left side. A multiple linear regression analysis was performed to identify any potential predictors of COx values. Hemoglobin (Hb), PCO_2_, flow, DO_2_ index (DO_2_i), Ο_2_ extraction ratio (O_2_ER), and perfusion ratio (PR) were included in the analysis. Significant equations were found on both sides. Predicted COx left was equal to 5.8 - 11.04O_2_ER - 0.04Hb (p=0.001, R^2^= 0.15). Predicted COx right was equal to 3.06 - 0.3flow - 6.8O_2_ER -0.03Hb + 0.02PCO_2_ + 0.004DO_2_i(p=0.03, R^2^=0.13). Targeting physiological perfusion and monitoring perfusion during CPB may have an additional impact on cerebral autoregulation and should be studied further.

## Introduction

Neurological complications remain a leading cause of morbidity associated with cardiac surgery. Impaired cerebral autoregulation and tissue malperfusion have been identified as pathophysiologic mechanisms associated with neurological adverse events [1]. Impaired cerebral autoregulation is linked to less tolerability of hemodynamic changes that accompany cardiac surgery. The cerebral oximetry index (COx) is the correlation coefficient of invasive mean arterial blood pressure (ABP) and cerebral oximetry (rSO_2_). Near-infrared spectroscopy may provide information on tissue malperfusion depicted in the frontal lobe [2]. COx values close to zero are considered to express functional autoregulation [3]. Our aim is to present data from cerebral oximetry and autoregulation index in a high-risk patient for neurological complications operated for aortic valve replacement under minimally invasive extracorporeal circulation (MiECC) and investigate their correlation with in-line metabolic indices of perfusion during cardiopulmonary bypass (CPB).

## Case presentation

This case report describes a 58-year-old female presenting for elective aortic valve replacement due to serious stenosis (Vmax = 4.6 m/s, mean gradient = 49 mmHg, and aortic valve area 0.9 cm). Her medical history was significant for multiple ischemic strokes, epilepsy, and heavy smoking. Magnetic resonance angiography was assessed by a multidisciplinary team consisting of vascular surgeons and radiologists who revealed stenosis of the brachiocephalic artery resulting in decreased flow of the corresponding vessel which extended to the initial carotid and subclavian artery. She was under medication with acetylsalicylic acid and carbamazepine.

Induction to general anesthesia was performed with target-controlled infusion (TCI) of propofol 1.5-2.5 mcg/ml and remifentanil 4-7 ng/ml along with a bolus dose of 3 mcg/kg fentanyl. Tracheal intubation was facilitated with 0.6 mg.kg-1 rocuronium. The maintenance of anesthesia was achieved with TCI of propofol. Standard monitoring, invasive blood pressure through a left side radial artery, depth of anesthesia (Bispectral Index, Medtronic), and rSO_2_ (INVOS 5100, Covidien-Medtronic Inc.) were applied. COx (ICM Software, University of Cambridge, Cambridge, UK) was monitored during the whole intraoperative period. During CPB under MiECC, continuous in-line perfusion data (System M, Spectrum Medical, FortMill, SC, USA) including hemoglobin (Hb), PCO_2_, flow, DO_2_ index (DO_2_i), carbon dioxide production (VCO_2_i), Ο_2_ extraction ratio (O_2_ER) and perfusion ratio (PR, corresponding to DO_2_i/ VCO_2_i) were recorded. Intraoperative management was implemented according to the published strategy for physiologic perfusion [4].

SPSS Statistics 23.0 (IBM Corp., Armonk, NY) was used for analysis. Continuous data are reported as mean and standard deviation. Multiple linear regression was employed to identify any independent predictors of the COx on both sides.

**Figure 1:**
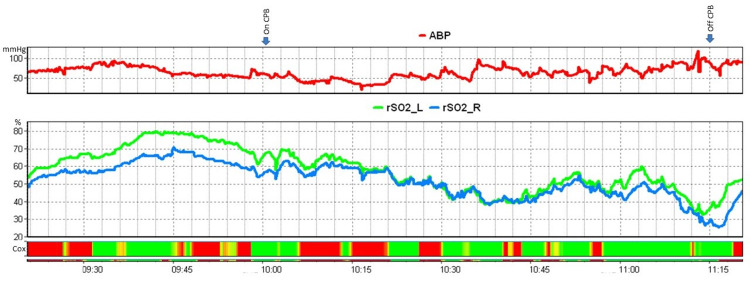
Intraoperative Course of Mean Arterial Blood Pressure (ABP) and Cerebral Oximetry (rSO2). The green area denotes intervals when cerebral oximetry index (COx) < 0.3. rSO_2__L: rSO2 left; rSO2_R: rSO2 right; CPB: cardiopulmonary bypass

Baseline rSO_2_ values were obtained before induction and were below 50 on both sides (rSO_2_ left=48, rSO_2_ right=40). We divided the intraoperative period into three phases; the interval from the anesthesia induction until the initiation of CPB set as the pre-CPB phase, the period of CPB, and the phase after weaning from CPB which included the immediate post-bypass period until the end of the procedure or post-CPB. A descriptive analysis of the rSO2, COx, and invasive ABP is provided in Table 1 (see Figure 1). A type IV MiECC circuit was used and the CPB duration was 71 minutes. Data were recorded at 10s intervals. As computed by the ICM software, the ABP lower limit of autoregulation was 44mmHg on both sides and the upper Llmit was 98mmHg on the right and 107mmHg on the left side. Regarding the CPB period, we performed a multiple linear regression analysis to identify any potential predictors of COx values. Possible predictors included DO_2_i, O_2_ER, PCO_2_, vCO_2_i, Hb, flow, and PR (Table 2). On both sides, significant equations were found. Predicted COx left was equal to 5.8 - 11.04O_2_ER - 0.04Hb (p=0.001, R^2^= 0.15). Predicted COx right was equal to 3.06 - 0.3flow - 6.8O_2_ER -0.03Hb + 0.02PCO_2_ + 0.004DO_2_i (p=0.03, R^2^=0.13).

**Table 1:** Intraoperative Parameters. COx: cerebral oximetry index; ABP: arterial blood pressure; rSO_2_: cerebral oximetry; L: left; R: right

	Pre-CPB	CPB	Post- CPB
COx L	0.01 ± 0.4	-0.1 ± 0.5	-0.2 ± 0.5
COx R	-0.01 ± 0.4	0.02 ± 0.5	-0.1 ± 0.5
ABP, mmHg	65 ± 12	60 ± 14	70 ± 13
rSO_2_ L, %	64 ± 10	52 ± 8	52 ± 6
rSO_2_ R, %	51 ± 12	49 ± 6	46 ± 7

**Table 2:** In-line Cardiopulmonary Bypass Metabolic Indices. Data are presented as median and interquartile ranges vCO2i: carbon dioxide production index; PCO_2_: partial pressure of carbon dioxide; DO_2_i: oxygen delivery index

Parameter	median (IQR)
flow, L/min	4.43 (0.37)
vCO_2_i, mL/min/m^2^	86 (17)
Perfusion ratio	4.28 (1.09)
O_2 _Extraction Ratio	0.15 (0.01)
Hemoglobin, mg/dl	10.2 (0.5)
PCO_2_, mmHg	43 (2.8)
DO_2_i, mL/min/m^2^	363 (49)

No delirium or any other neurologic complication was recorded after extubation even though extubation was delayed until 18 hours postoperatively due to the development of pneumothorax and respiratory compromise.

## Discussion

Impairment of cerebral autoregulation and low baseline near-infrared spectroscopy (NIRS) values have been associated with postoperative delirium [5]. Also, in a randomized controlled trial, baseline cerebral oximetry values ≤50% were linked to a four-fold increase in the occurrence of delirium [6]. Hori et al. point out that determining the blood pressure limits of autoregulation may be beneficial in preventing delirium as malfunctioning autoregulation occurs often intraoperatively. Tight control of blood pressure within these limits during surgery has been shown to lower the rate of delirium events as mean arterial blood pressure is a major determinant of cerebral perfusion pressure [7].

However, individualized measures should not be restricted to blood pressure, but extend to other important metabolic markers of perfusion. Goal-directed perfusion during CPB focuses on metabolic targets such as pump flow, oxygen delivery, and carbon dioxide production to optimize patient outcomes. Low DO_2_ has been proven to be a cause of postoperative kidney injury [8]. Parallel to this, delirium has been correlated with the duration of mixed venous oxygen saturation values lower than 75% [9]. The importance of monitoring in-line metabolic indices has also been incorporated in the CPB guidelines (Class IIa, level of evidence B) as a tool to evaluate the adequacy of pump flow rate [10].

Our patient's low baseline NIRS combined with the history of cerebrovascular disease predisposes the patient to neurologic complications. Cerebral autoregulation had not been correlated before with metabolic indices measured during CPB. In this high-risk case, we demonstrated the significant effect of pump flow, hemoglobin, ventilation, and oxygen extraction ratio and delivery on COx based on recordings from in-line monitoring. The smooth postoperative course of this patient may be attributed to our strategy for physiologic cardiac surgery, associated with less inflammation and avoidance of hemodilution [4, 11]. In our department, the use of MiECC encompasses per se goal-directed perfusion which may contribute to attenuation of physiologic disturbances during cardiac surgery, especially in high-risk patients. Continuous monitoring enables preemptive measures to prevent deviations from metabolic targets. This poses a challenge to investigate the effects of incorporating other parameters, except for blood pressure and cerebral oximetry, on postoperative neurological complications. According to our findings in this high-risk patient, in-line metabolic indices of perfusion are independent predictors of cerebral autoregulation during cardiac surgery.

A limitation of this report is the lack of ICU monitoring of COx due to technical issues with software compatibility. Further studies need to be designed to investigate the clinical benefit of this strategy on neurological outcomes after cardiac surgery. Our team intends to recruit more patients to validate this concept.

## Conclusions

In conclusion, metabolic indices of perfusion may affect cerebral autoregulation in high-risk cardiac surgery patients. In-line monitoring may minimize physiological derangements and maintain cerebral autoregulation during CPB. MiECC focuses on goal-directed perfusion as a strategy to monitor and prevent such derangements in view of limiting complications. Our findings may attract researchers to question the impact of targeting metabolic parameters on preserving cerebral autoregulation during cardiac surgery on high-risk patients.
